# Exosomal miR-423-5p mediates the proangiogenic activity of human adipose-derived stem cells by targeting Sufu

**DOI:** 10.1186/s13287-019-1196-y

**Published:** 2019-03-21

**Authors:** Fen Xu, Qinqin Xiang, Jiuzuo Huang, Qianlong Chen, Nanze Yu, Xiao Long, Zhou Zhou

**Affiliations:** 1 Center of Laboratory Medicine, State Key Laboratory of Cardiovascular Disease, Fuwai Hospital, National Center for Cardiovascular Diseases, Chinese Academy of Medical Sciences and Peking Union Medical College, No.167 North Lishi Road, Xicheng District, Beijing, 100037 People’s Republic of China; 2 Division of Plastic Surgery, Peking Union Medical College Hospital, No.1 Shuaifuyuan Wangfujing Dongcheng District, Beijing, 100730 People’s Republic of China

**Keywords:** Human adipose-derived stem cells, Exosomes, Angiogenesis, miRNAs, RNA sequencing

## Abstract

**Background:**

Human adipose-derived stem cells (hADSCs) are an important source of cells for regenerative medicine. Evidence of extensive interactions with the surrounding microenvironment has led researchers to focus more on hADSCs as activating agents of regenerative pathways, rather than simply replacing damaged cells. Several studies have found that functional miRNAs can be packaged into exosomes and transferred from donor cells into recipient cells, indicating that transported miRNAs may be a new class of cell-to-cell regulatory species. The aim of the present study was to evaluate whether the exosome-derived miRNAs secreted by hADSCs are capable of influencing angiogenesis, a key step in tissue regeneration.

**Methods:**

Exosomes were purified from hADSCs followed by the characterization of their phenotype and angiogenic potential in vitro. RNA sequencing was performed to detect the miRNAs that were enriched in the hADSC-derived exosomes. A miRNA-mimic experiment was used to detect the key miRNAs in the proangiogenic activity of hADSC-derived exosomes.

**Results:**

Exosomes isolated from hADSCs were characterized as round membrane vesicles with a size of approximately 100 nm and were positive for CD9 and flotillin. The exosomes were internalized by primary human umbilical vein endothelial cells (HUVECs) and stimulated HUVEC proliferation and migration. Remarkably, the exosomes promoted vessel-like formation by HUVECs in a dose-dependent manner, and their maximum activity (10 μg/mL) was comparable with that of 5% FBS. The RNA-seq bioinformatics analysis predicted 1119 gene targets of the top 30 exosomal miRNAs in Gene Ontology (GO) analysis and Kyoto encyclopedia of genes and genomes (KEGG) pathway analysis, and the pathway involved in the angiogenesis was among the top KEGG pathways. Moreover, intact miR-423-5p was further demonstrated to be transferred into HUVECs via exosomes and to exert its angiogenic function by targeting Sufu.

**Conclusions:**

Exosomal miR-423-5p mediated the proangiogenic activity of hADSCs by targeting Sufu, which may contribute to the exploitation of exosomes from hADSCs as a therapeutic tool for regenerative medicine.

**Electronic supplementary material:**

The online version of this article (10.1186/s13287-019-1196-y) contains supplementary material, which is available to authorized users.

## Introduction

Mesenchymal stem cells (also referred to as mesenchymal or multipotent stromal cells, MSCs) were first discovered in the bone marrow and have subsequently been found in other tissues (umbilical cord blood, scalp tissue, amniotic fluid, placenta, adipose tissue, etc.) of the body [1]. MSCs show great promise in a wide array of therapeutic applications due to their reported regenerative potency. The use of MSCs for regenerative medicine in humans has been proposed. Currently, they are being clinically investigated against an increasingly wide spectrum of disease indications, particularly for tissue repair and regeneration [2]. The cell therapy field has recently witnessed a significant expansion of the uses of MSCs in clinical trials as indicated by an approximately threefold increase in the number of Investigational New Drug submissions to the FDA for MSC-based products between 2006 and 2012 [3] and the initiation of approximately 235 MSC clinical trials worldwide (http://www.clinicaltrials.gov/, queried in November 2018).

The use of MSCs for tissue regeneration was initially based on the hypothesis that these cells infiltrated the injured tissue and differentiated into tissue-specific functioning cells [4–6]. However, the observation that only small proportions of transplanted MSCs survive and integrate into the damaged host tissues has highlighted the possibility that alternative mechanisms might exist. Indeed, evidence of extensive interaction with the surrounding microenvironment has led researchers to focus more on MSCs as activating agents of regenerative pathways rather than simply replacing damaged cells [7]. Recent discoveries suggest that exosomes released by MSCs may also be important in the physiological function of these cells [8].

Exosomes are small vesicles between 50 and 150 nm in diameter that are secreted by most cell types and have been described as a new mechanism for cell-to-cell communication [9–11]. Their ability to interact with neighboring cells and/or with distant cells through biological fluids makes them a medium through which intercellular exchange of information can occur. Several studies have shown that MSC-derived exosomes possess a similar therapeutic efficacy to donor cells in tissue repair and possibly anti-cancer therapy [12–15]. These results suggest that the development of novel exosome-based therapeutic approaches might reduce the obstacles and risks associated with the transplantation of intact native or engineered stem cells.

Exosomes derived from MSCs have been particularly studied for their beneficial effects on angiogenesis, a key step in tissue regeneration, but their mechanism of action remains unclear [16–18]. miRNAs are small (20–22 nt) noncoding RNAs that suppress protein translation by binding to target mRNAs, thereby reducing the stability of these targets and/or inhibiting translation. A specific class of miRNAs, angio-miRNAs, has been shown to be pivotal modulators of vascular development and angiogenesis [19–21]. Growing evidence suggests that functional miRNAs could be packaged into exosomes and transferred from donor cells into recipient cells, which indicates that transported miRNAs may be a new class of cell-to-cell regulatory species [22]. The aim of the present study was to evaluate whether exosomes secreted by human adipose-derived stem cells (hADSCs) are capable of influencing angiogenesis and to determine whether the miRNAs of these exosomes play a crucial role in the angiogenic process.

## Materials and methods

### hADSC culture and identification

Adipose tissue samples were obtained with informed donor consent from people who had undergone liposuction. The tissue sampling procedure was approved by the Ethics and Welfare Committee of Chinese Academy of Medical Sciences and Peking Union Medical College (Protocol number 2012-034; approved 12 September 2012). The samples were washed with Hank’s balanced salt solution (HBSS; Gibco). The adipose tissue samples were digested in 0.1% Collagenase Type I (1 mL for each 1 mL of tissue, Gibco) at 37 °C for 30 min with shaking every 5 min. After digestion, an equal volume of HBSS was added. The cell suspension was filtered through 100-μm filters (BD Falcon) to remove the solid aggregates. The samples were subsequently centrifuged at 1500 rpm for 5 min at 4 °C to obtain a pellet containing the stromal vascular cell fraction. The centrifugation step was repeated. The cells were incubated at 37 °C with 5% CO_2_ for 72 h in culture dishes with complete basal medium that consisted of Mesenchymal Stem Cell Basal Medium (MSCBM; DAKEWEI), 5% UltraGRO-Advanced (GMP Grade, Helios), 100 U/mL penicillin, and 100 mg/mL streptomycin. After 1 day, the nonadherent cells were removed by two to three washes with HBSS, and medium changes were performed every 2 days thereafter. The cells were expanded until they achieved 80% confluence. The cell morphology was monitored using an inverted microscope. Passage 3 cells were used for flow cytometric analysis and differentiation assays.

For the flow cytometric analysis, 5 × 10^5^ ADSCs (in 100 μL PBS) were incubated with various fluorescently labeled monoclonal antibodies (anti-human CD45-PE, anti-human CD31-PE, anti-human HLA-DR-FITC, anti-human CD29-PE, anti-human CD44-PE, anti-human HLA-A, B, C-PE, anti-human CD90-FITC, anti-human CD105-FITC, anti-human CD13-FITC, Biolegend) and incubated in the dark at 2–8 °C for 30 min. After washing twice with PBS, the cells were resuspended in 300 μL phosphate-buffered saline (PBS) and analyzed using a Calibur flow cytometer (Mindray).

For the multidirection differentiation assay, hADSCs were induced with specific adipogenic, osteogenic, or chondrogenic inductive media (Lonza) for 3 weeks. Then, the cells were identified with Oil Red O, Alizarin Red, or Alcian Blue staining, respectively.

### Exosomes isolation and identification

Exosomes were obtained from the supernatants of hADSCs (at passages 3 to 6) after 2 days culture in DMEM. The culture supernatant was centrifuged at 2000*g* for 10 min to remove the dead cells. The cell-free supernatant was then centrifuged at 10,000*g* for 30 min to remove debris. The debris-free supernatant was subjected to ultracentrifugation (Beckman optima LE-80 K) at 100,000*g* for 70 min at 4 °C, washed in 0.1 M PBS, pH 7.3, and subjected to a second ultracentrifugation step under the same conditions. Exosomes from 1 × 10^7^ cells were resuspended in 200 μL PBS. To visualize the exosomes using electron microscopy, carbon-coated Formvar film grids were placed on 5 mL of an exosome suspension for 20 min and washed three times with 0.15% glycine in PBS, and once with 0.1% bovine serum albumin in PBS. The vesicles were fixed in 1% glutaraldehyde in PBS for 5 min and washed twice with PBS. After washing with distilled water, the grids were placed on a drop of phosphotungstic acid for 5 min and then air-dried. The exosomes were visualized using a Tecnai 12 transmission electron microscope (FEI, Hillsboro, OR). In addition, the NanoSight (Malvern, UK) was used to evaluate the exosome size distribution. The surface markers flotillin and CD9 (Cell Signaling Technology) were detected by Western blotting, and GAPDH (Cell Signaling Technology) was used as an internal reference for cell lysates.

### Exosome uptake

Exosomes were labeled with the red fluorescent dye PKH26 (Sigma) for tracking purposes. The exosomes from 10 million cells were resuspended in 180 μL of PBS with 20 μL of 1:50 diluted PKH26 (in Diluent C). After 3 min of incubation at room temperature (RT), 3.8 mL of exosome-free medium was added to terminate the labeling reaction, and then, the exosomes were harvested and washed twice with PBS by centrifugation (100,000*g* for 70 min). The exosomes were resuspended in FBS-free DMEM (10 μg L^−1^), added to a subconfluent layer of human umbilical vein endothelial cells (HUVECs) in a 24-well plate (500 μL per well), and incubated for 21 h at 37 °C. Every 3 h, one of wells was washed twice with PBS, fixed with 4% paraformaldehyde for 30 min at RT, and stained with DAPI (4,6 diamidino-2-phenylindole). Then, cells were analyzed using a Zeiss confocal microscope (ZEISS LSM780).

### Isolation of human umbilical vein endothelial cells (HUVECs)

The umbilical cords were obtained with informed donor consent following healthy births. The umbilical veins were perfused with HBSS (Gibco) that was supplemented with 200 U/mL penicillin and 200 mg/mL streptomycin. One end of each umbilical vein was closed with a hemostat, and 0.1% type I collagenase in HBSS was introduced to the lumen. The open end of the umbilical vein was clamped shut with another hemostat and incubated at 37 °C for 30 min. After a gentle massage of the umbilical cord, the endothelial cell-collagenase suspension was poured off and diluted 1:1 with HBSS. The vessel lumen was washed twice with HBSS, and the combined cell suspension was centrifuged at 1000 rpm for 10 min. The cell pellet was washed once with HBSS, suspended in 10 mL complete ECM medium (Sciencell), seeded in a 10 cm petri dish, and incubated at 37 °C, with 5% CO_2_. The HUVECs were not used for experiments beyond passage seven. The HUVECs were identified by immunofluorescent staining for an endothelial cell marker, vWF.

### Vessel-like formation assay

HUVECs (3 × 10^4^ cells per well) were seeded onto Matrigel-coated wells in a 24-well plate and cultured in ECM medium without FBS in the presence of exosomes at various concentrations: 0, 2.5, 5, and 10 μg/mL. As a positive control, 5% FBS was used. After incubation for 24 h, phase-contrast images were recorded and the total length of the network structures was measured using ImageJ (https://imagej.nih.gov/ij/). For evaluating the role of miR-423-5p on tube formation ability of endothelia cell, the HUVECs transfected with miR-423-5p for 48 h was seeded onto Matrigel-coated wells in a 48-well plated and cultured in ECM complete media for 12 h. The total length per field was calculated in five random fields and expressed as a ratio to the respective control.

### Scratch assay

Endothelial cell migration was assessed by scratching a confluent layer of HUVECs in a 24-well plate using a 20 to 200 μL pipette tip. The loose cells were removed by washing with PBS, and 200 μL test medium (with/without exosomes) was added, followed by incubation at 37 °C. Images were recorded at 0 h and 24 h. For evaluating the role of miR-423-5p on endothelial cell migration, the HUVECs were transfected with miR-423-5p for 48 h, followed by the scratch assay as above mentioned. Images were recorded at 0 h and 12 h. The reduction in the wound area was determined using Zeiss software (version 3.0; Media Cybernetics).

### Cell proliferation assays

HUVECs were suspended in complete ECM medium and seeded in 24-well plate at 2 × 10^4^ cells per well. After 24 h, the cells were cultured with FBS-free ECM for 6 h. Then, exosomes were added (5 μg/500 μL/well) for 24 h. Simultaneously, 5% FBS ECM and FBS-free ECM were used as positive and negative controls. DNA synthesis was detected as the incorporation of 5-ethynyl-2′-deoxyuridine (EdU; RiboBio) into the cellular DNA, following the manufacturer’s instructions.

### Exosome small RNA extraction, library construction, sequencing and bioinformatics analysis

To conduct the small RNA sequencing, the exosomes were isolated using Ribo Exosome Isolation Reagent (RiboBio), and the size distribution was determined using a ZETASIZER Nano series-Nano-ZS (Malvern, England). Total cellular RNA was extracted using Trizol (Invitrogen) according to the manufacturer’s instructions. Library construction was performed using NEBNext® Multiplex Small RNA Library Prep Set for Illumina® (Illumina, USA). RNA sequencing was performed on an Illumina HiSeq2500 sequencer. The raw reads obtained by sequencing were initially filtered by removing the adapters at both ends of the reads, removing low-quality reads, decontamination, etc., resulting in a clean sequence (clean reads). The clean reads were mapped to the reference genome using BWA software. The filtered reads were aligned with the authoritative miRNA/rRNA/tRNA/snRNA/snoRNA database to annotate known ncRNAs, including miRNA (miRBase version 21), rRNA, tRNA, snRNA, and snoRNA (Rfam12.1). The clean reads were aligned with all mature miRNAs in the miRBase database (version 21), and the expression levels were normalized to the number of reads per million (RPM). The Pearson correlation coefficient (r) was used to measure the linear correlation between two variables. The expression level of the top 30 miRNAs accounted for more than 80% of the total miRNA expression levels, therefore, we selected the top 30 miRNAs for target prediction using TargetScan, miRDB, miRTarBase, and miRWalk, and the intersection targets predicted by multiple databases were listed as candidate target genes for miRNA. Gene Ontology (GO) analysis was used to annotate each gene and calculate the most significant functions of a particular gene set by hypergeometric distribution statistical analysis. Biological Pathway analysis was based on the Kyoto encyclopedia of genes and genomes (KEGG) biological pathway database.

### RNA extraction, reverse transcriptase (RT)-PCR and real-time RT-PCR

Total cellular RNA was extracted using a miRNeasy Mini Kit (Qiagen, Dusseldorf, Germany) according to the manufacturer’s instructions. For miRNA detection, RNA was transcribed into cDNA using the miScript II RT Kit (Qiagen, Dusseldorf, Germany). Quantification of the RNA levels for 30 selected miRNAs for qPCR validation (Table 1) was achieved by quantitative RT-PCR using SYBR Green PCR Kit (Qiagen, Dusseldorf, Germany) and an ABI ViiA7 system. The data were normalized to the expression of U6. For the measurement of suppressor of fused (Sufu) mRNA, the RNA was transcribed into cDNA using the PrimeScrip™ Reverse Transcriptase system (Takara, Kusatsu, Japan), and quantification of the RNA levels for Sufu was achieved by quantitative RT-PCR using an ABI ViiA7 system and PowerUp SYBR Green Master Mix (Applied Biosystems, USA). The data were normalized to the expression of GAPDH. Each reaction was run in triplicate, and the data were analyzed according to the threshold cycle (Ct) method.

### miR-423-5p mimic transfection

The HUVECs were seeded onto cell culture plates at a density of 5000 cells per square centimeter. After culture for 24 h, the cells were transfected for 48 h with the miR-423-5p mimic (10 nM) using Lipofectamine RNAiMAX (Thermo Fisher, USA). All transfection experiments were performed in triplicate. Subsequently, RNA and protein were extracted to determine the changes of Sufu expression.

### Statistical analysis

All data are presented as the means ± SD. To represent the relative gene expression levels, the mean value of the vehicle control group was defined as 100% or 1. Two-tailed unpaired Student *t* tests and analyses of variance were used for the statistical evaluation of the data. SPSS (version 17.0; IBM, Armonk, New York) was used for data analysis. A *p* value < 0.05 was considered to be significant.

## Results

### Characterization of hADSCs

**Fig. 1:**
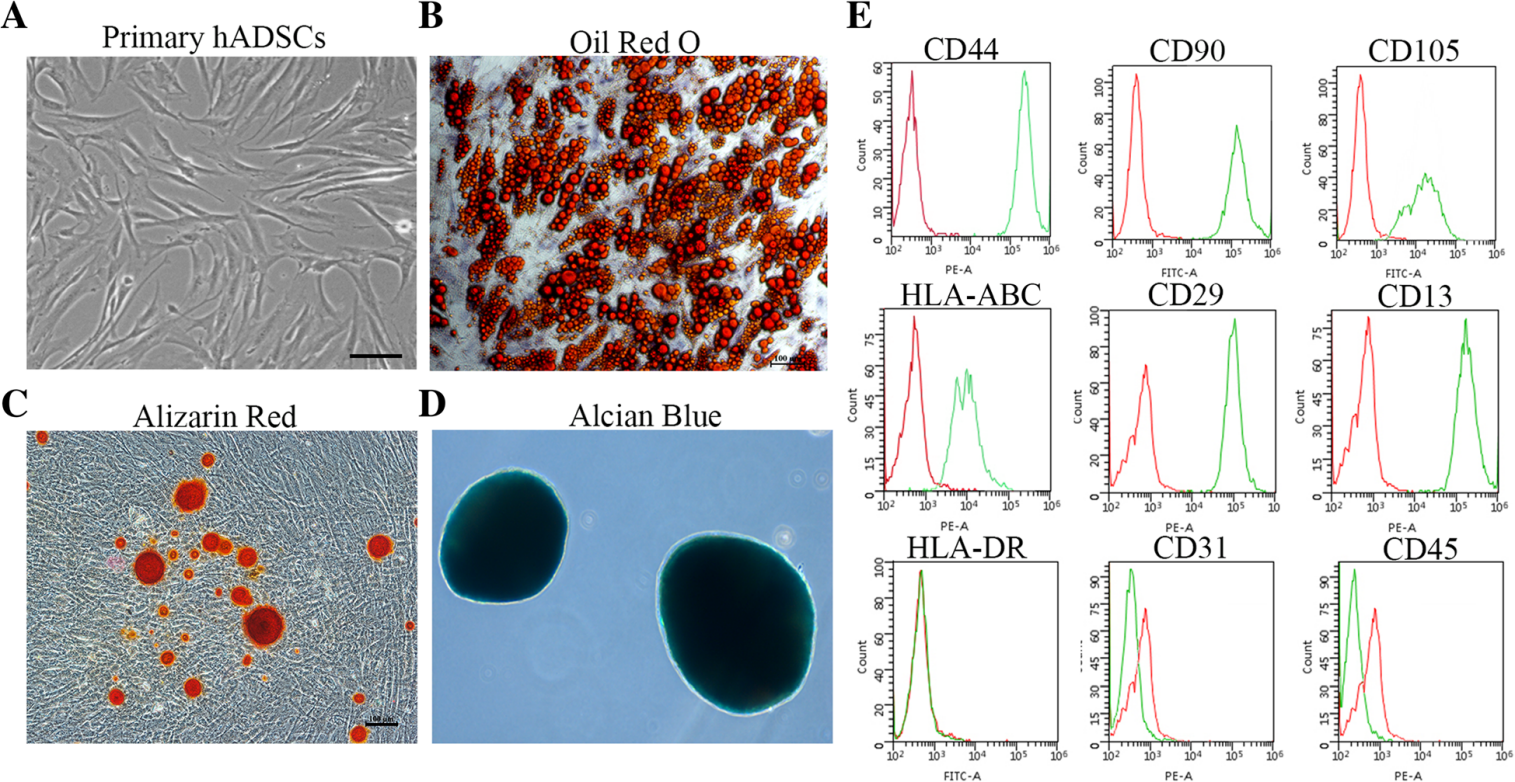
Characterization of hADSCs. **a** Representative photos of the morphology of hADSCs. Bar = 100 μm. **b–d** The adipogenic, osteogenic and chondrogenic ability of hADSCs were identified by Oil red O staining (**b**), Alizarin Red staining (**c**), and Alcian Blue binding (**d**), respectively. Bar = 100 μm. **e** Flow cytometric analysis identified the positive surface markers CD44, CD90, CD105, HLA-ABC, CD29, and CD13 and the negative surface markers HLA-DR, CD31, and CD45. The green line represents the signal of the antibody we detected, and the red line represents the signal of the isotype control

hADSCs were isolated from routine liposuction samples according to the description in the “Materials and methods” section. The cells were adherent and displayed the fibroblastic morphology that is typical of MSCs (Fig. 1a). Moreover, they were capable of self-renewal and multilineage differentiation. When exposed to appropriate induction medium, they could differentiate into adipogenic, osteogenic and chondrogenic cells, which were identified by Oil red O staining (Fig. 1b), Alizarin Red staining (Fig. 1c), and Alcian Blue binding (Fig. 1d), respectively. Furthermore, flow cytometric analysis showed that these cells expressed the surface markers that are characteristic of MSCs (positive for CD44, CD90, CD105, HLA-ABC, CD29, and CD13; negative for HLA-DR, CD31, and CD45) (Fig. 1e). Based on these results, the hADSCs possessed the common characteristics of MSCs.

### hADSC-derived exosomes are internalized by HUVECs

**Fig. 2:**
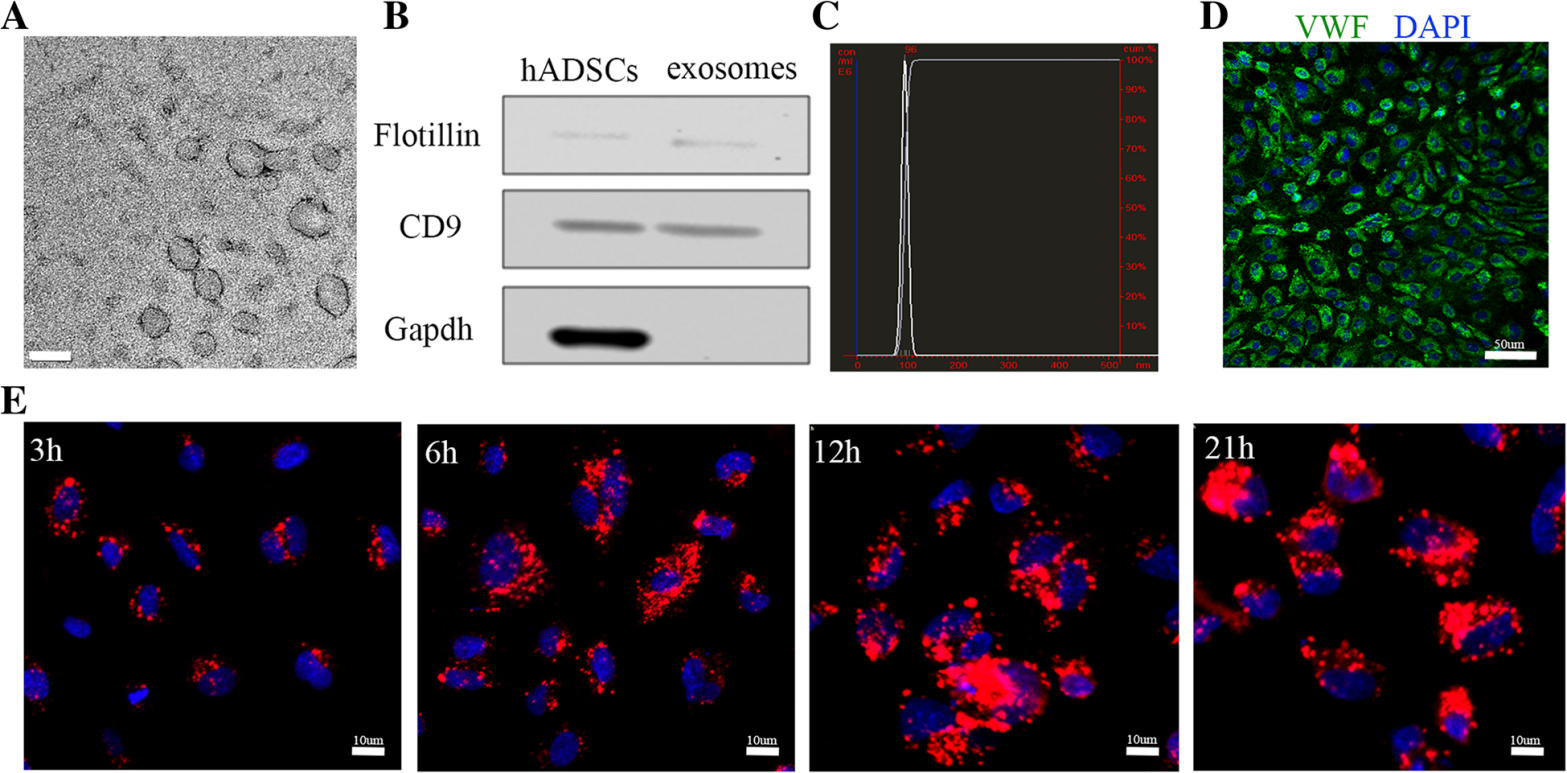
Identification of the hADSC-derived exosomes and their uptake by HUVECs. Identification and characterization of hADSC-derived exosomes by TEM (**a**), NanoSight (**b**), and Western blot detection of exosome surface markers CD9 and flotillin (**c**). Bar = 100 nm. **d** Immunofluorescence staining identification of HUVECs by an endothelial cell marker, vWF. Bar = 50 μm. **e** Representative photos of the HUVEC uptake of PKH26 labeled exosomes (10 μg/ml) at 3 h, 6 h, 12 h, and 21 h. Bar = 10 μm

Exosomes were collected from the hADSC culture media using classical ultracentrifugation. After removal of microvesicles using a 0.2-μm filter, the purified exosomes were obtained by ultracentrifugation at 100,000*g*. This exosome purification procedure was first validated using transmission electron microscopy. Electron microscopy of negatively stained exosomes revealed rounded membrane vesicles with a size of approximately 100 nm (Fig. 2a). Immunoblotting showed that CD9 and flotillin, two common exosomal proteins [23], were present in the exosome fraction and hADSC lysates, but GAPDH was detected exclusively in the cell lysates, which suggested that no cell debris was present in the exosomes (Fig. 2b). Further characterization of the exosomes including size measurement and quantification was performed using NanoSight analysis (NTA), which demonstrated the purity of the vesicles, with a peak size at 96 nm (Fig. 2c, Additional file 1: Figure S1). The size of the hADSC-derived exosomes was consistent with that previously reported for exosomes, which are 50–150 nm in diameter. The exosome yield per 10^6^ ADSCs within 2 days of starvation was 3–4 × 10^8^ particles as determined by NTA or 3–4 μg protein quantified by the bicinchoninic acid (BCA) method. Taken together, these experiments indicate that hADSCs secrete exosomes to the culture supernatant.

HUVECs were primary cell types generated ex vivo from isolated tissue. HUVECs were grown in ECM medium containing 5% fetal bovine serum (FBS); all media components were from ScienCell Inc. Under inverted microscopy, the HUVECs appeared to be a single homogeneous cell population that exhibited the cobblestone-like morphology of endothelial cells (Additional file 1: Figure S2). The endothelial phenotype of the HUVECs was further confirmed by immunofluorescence staining that demonstrated a high level of expression of vWF (an endothelial cell marker) (Fig. 2d). Collectively, these results demonstrate that the HUVECs had both the morphology and function of endothelial cells. Currently, it is been proposed that the cells internalize exosomes either by fusion with the plasma membrane or via endocytosis [24]. Caveolin-mediated endocytosis in epithelial cells has been described [25]. To observe the internalization of the exosomes by HUVECs, the exosomes were stained with PKH26 (a red fluorescent cell linker), which labeled the membrane phospholipids. Selected concentrations of PKH26-labeled exosomes were incubated with HUVECs for various times. The internalized exosomes were visualized as dots of red fluorescence around the HUVEC nuclei in a concentration- and time-dependent manner (Fig. 2e, Additional file 1: Figure S3). The findings support the ability of exosomes to influence cell behavior via autocrine and/or paracrine routes.

### Proangiogenic effect of exosomes derived from hADSCs

**Fig. 3:**
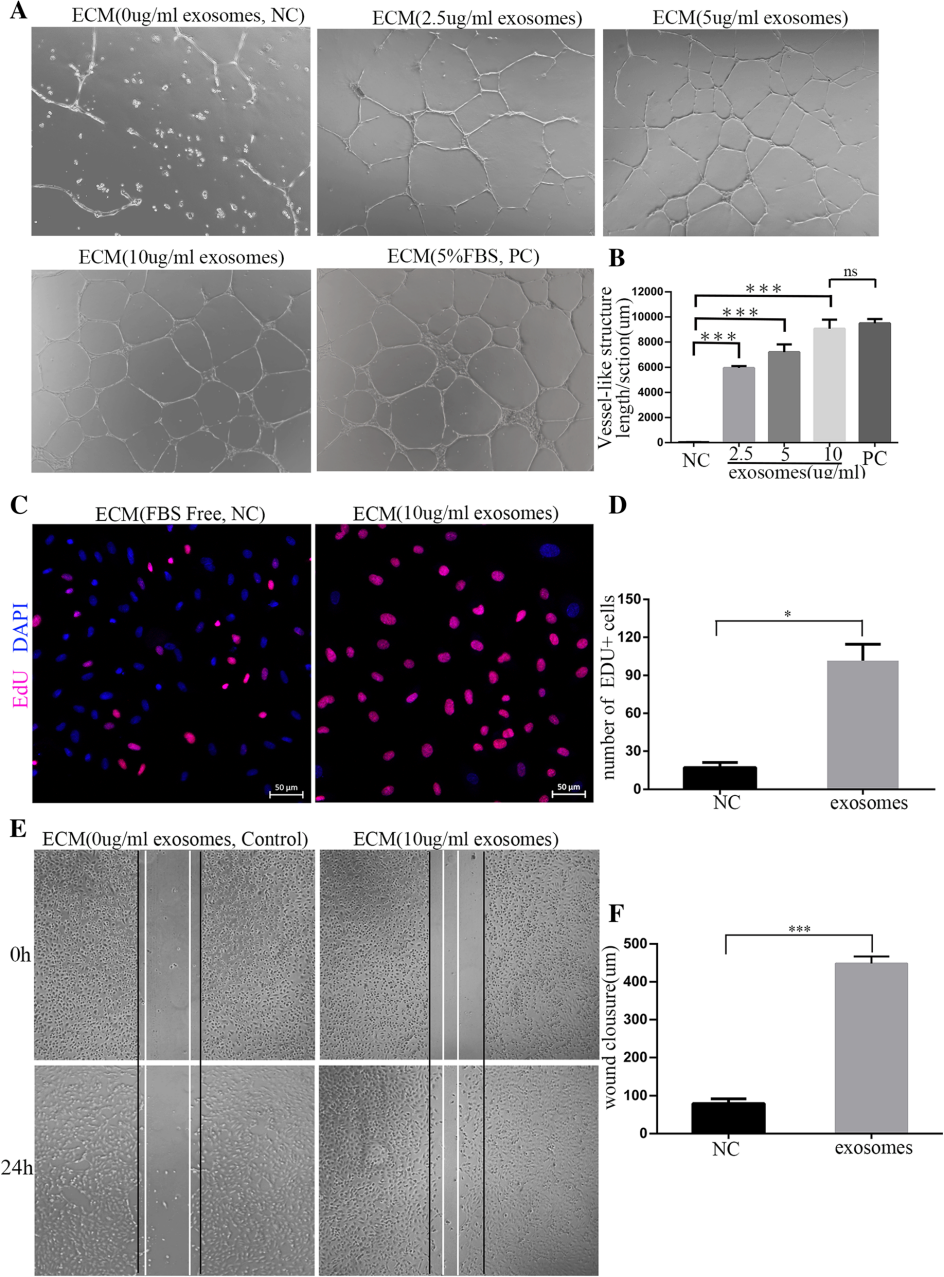
Proangiogenic effect of exosomes derived from hADSCs. **a** The vessel-like structure formation assay was used to assess the tube formation ability of HUVECs cultured on Matrigel Basement Membrane Matrix in exosome-conditional media (0, 2.5, 5, or 10 μg/mL) for 24 h, 5% FBS was used as a positive control. **b** The total length of the network structures was measured using the ImageJ analysis system. The total length per field was calculated in five random fields and expressed as a ratio to the respective control. **c** An EdU experiment was used to assess the proliferation ability of HUVECs cultured in exosome-conditioned media (10 μg/mL) for 24 h. Cells that were not treated with exosomes served as the control. Bar = 50 μm. **d** The statistical analysis of the number of EDU-positive cells. **e** Images of scratch assays recorded at 0 h and 24 h of culture of the HUVECs in exosome-conditioned media (10 μg/mL). **f** Wound closure determined using software

Angiogenesis is normally initiated after preliminary destabilization of pre-existing vessels, whereby the proliferation and migration of endothelial cells cause endothelial sprouting, canalization, and eventually stabilization of the vessel wall, which leads to the formation of new blood vessels [26]. To evaluate the effect of hADSC-derived exosomes on angiogenesis, the HUVEC vessel-like structure formation assay was first performed in vitro. HUVECs were cultured on Matrigel Basement Membrane Matrix in exosome-containing media (0, 2.5, 5, or 10 μg/mL) for 24 h. As a positive control, 5% FBS was used. The result showed that the hADSC-derived exosomes significantly promoted the formation of vessel-like structures by the HUVECs in a dose-dependent manner, and their maximum activity (at 10 μg/mL) was comparable to that of 5% FBS (Fig. 3a, b).

EdU uptake is an important technique for determining cell proliferation. When cultured in exosome-conditional media (10 μg/mL) for 24 h, the HUVECs significantly increased EdU uptake compared to those cells that were not treated with exosomes (Fig. 3c, d). This suggests that the exosomes derived from hADSCs are able to stimulate endothelial cell proliferation. Meanwhile, in the scratch assay, HUVEC migration was distinctly accelerated after incubation with exosomes (10 μg/mL) with many cells migrating into the gap within 24 h (Fig. 3e), and the wound closure was significantly greater in the exosome-treated samples than that in control (Fig. 3f). In the treated samples, the cells facing the wound edge exhibited a typical polarized and migrating morphology. In summary, hADSC-secreted exosomes are capable of enhancing endothelial cell migration.

### miRNAs enriched in hADSC-derived exosomes

**Fig. 4:**
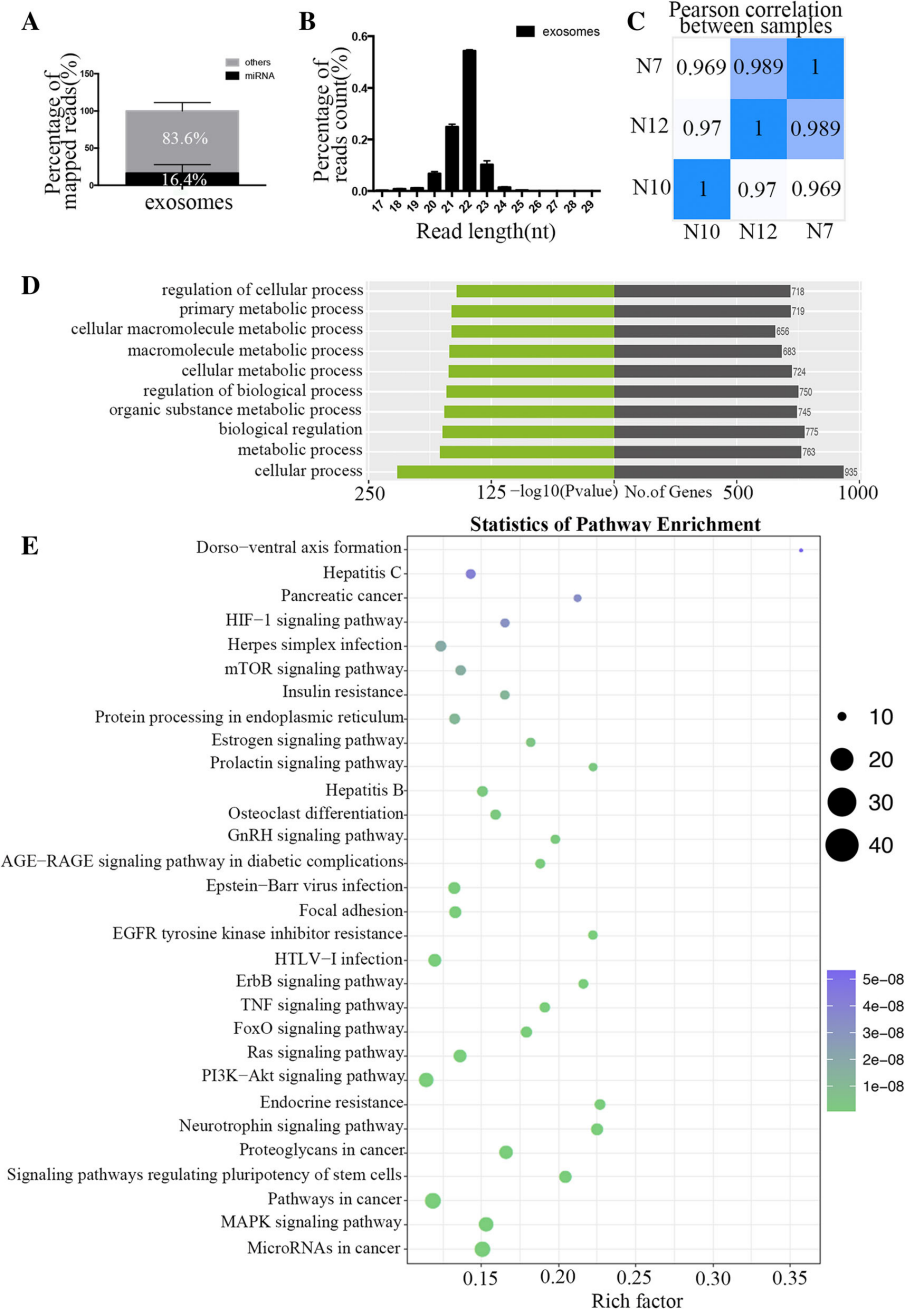
RNA-seq identified the miRNAs that are enriched in hADSC-derived exosomes and the predicted GO Terms and KEGG pathways that were targeted. **a** The average proportion of annotated miRNAs in the clean reads. **b** The size distribution of miRNAs reads was between 17 and 29 nt with a peak of 22 nt. **c** The Pearson correlation of three repeats was greater than 96.9%. The Gene ontology (GO) analysis (**d**) and Kyoto encyclopedia of genes and genomes (KEGG) pathway analysis (**e**) of 1119 gene targets of the 30 most abundant miRNAs in the exosomes

miRNAs participate in a diverse range of regulatory events via the regulation of genes that are involved in the control of processes such as angiogenesis, a key pathway in placental vascular development in pregnancy. This suggests an important role of the miRNAs that regulate angiogenesis. The mechanistic roles of some miRNAs in modulating endothelial cell (EC) function in physiology and in disease have been described. To explore whether the miRNAs play a key role in the proangiogenic effect of hADSC-derived exosomes, we isolated the exosomes from the hADSC culture media of three patients and performed RNA sequencing to evaluate their miRNAs expression. On average, we generated 19.1 million reads for each sample, and the Phred quality score greater than or equal to 30 (Q30) was 92.7%, which provided sufficient data quality for bioinformatic analysis. After filtering the raw data, 14.2 million reads (74.3%) were used for further analysis. Approximately 93.4% of the reads were mapped to the reference genome using BWA software, and the average proportion of annotated miRNAs was 16.4% (Fig. 4a). The size distribution was between 17 and 29 nt with a peak at 22 nt (Fig. 4b), and the expression profiles had a high correlation of greater than 96.9% in three repeats (Fig. 4c), which indicates the high stability of the exosome miRNAomes.

Overall, 845 miRNAs were detected in at least two of the samples, which accounted for 32.7% of the 2588 mature miRNAs in the miRBase (version21). The expressed miRNAs are listed in the Table 1 according to the value of mean RPM. The top 30 miRNAs (Table 1) account for 84.6% of the total reads on average and had similar profiles in three samples.

**Table 1:** Abundant miRNAs (top 30) expressed in exosomes derived from hADSCs

miRNA ID	Mean RPM ± SD	miRNA ID	Mean RPM ± SD
hsa-miR-148a-3p	170,404.48 ± 29,222.33	hsa-miR-486-5p	14,989.46 ± 2064.81
hsa-miR-320a	82,888.72 ± 17,890.58	hsa-miR-99a-5p	13,733.29 ± 1214.27
hsa-miR-146a-5p	74,802.90 ± 22,662.03	hsa-miR-143-3p	12,575.67 ± 1444.55
hsa-miR-151a-3p	54,850.96 ± 4946.64	hsa-miR-193a-5p	11,423.10 ± 2121.78
hsa-miR-423-5p	48,281.34 ± 5100.82	hsa-let-7a-5p	10,878.81 ± 2320.94
hsa-miR-100-5p	46,340.02 ± 5168.61	hsa-miR-10a-5p	10,120.11 ± 2273.86
hsa-let-7i-5p	38,749.16 ± 4501.18	hsa-miR-10b-5p	9288.26 ± 2230.98
hsa-miR-24-3p	33,189.69 ± 6788.42	hsa-let-7f-5p	8236.07 ± 1817.88
hsa-miR-199b-3p	30,780.60 ± 5523.64	hsa-miR-25-3p	7709.57 ± 1253.82
hsa-miR-199a-3p	26,382.55 ± 2146.59	hsa-miR-125b-5p	7399.15 ± 1275.36
hsa-miR-21-5p	24,935.23 ± 3296.01	hsa-miR-26a-5p	6921.62 ± 1751.13
hsa-miR-221-3p	21,532.58 ± 1245.14	hsa-miR-30d-5p	6743.80 ± 1804.46
hsa-let-7b-5p	20,600.91 ± 1816.55	hsa-miR-181a-5p	6652.67 ± 1823.19
hsa-miR-584-5p	16,941.48 ± 1749.60	hsa-miR-134-5p	6200.34 ± 2097.93
hsa-miR-92a-3p	16,385.24 ± 2363.62	hsa-miR-128-3p	5670.56 ± 1935.77

### GO terms and KEGG pathways for targets of hADSC-derived exosome-enriched miRNAs

To explore the functional categories of the miRNAome in exosomes derived from hADSCs, we predicted the targets of the top 30 most abundant miRNAs using miRDB, miRWalk, TargetScan and miRTarBase. In total, 1119 gene targets of the top 30 miRNAs in exosomes were first examined using GO analysis. Based on the biological processes (BP) of the gene ontology classification by GO analysis, the significant GO Terms were mainly associated with cellular processes, metabolic processes, and biological regulation, including “organic substance metabolic process,” “regulation of biological process,” “cellular metabolic process,” “macromolecule metabolic process,” “cellular macromolecule metabolic process,” “primary metabolic process,” and “regulation of cellular process” (Fig. 4d).

To further explore the pathways in which the targets were involved, Kyoto encyclopedia of genes and genomes (KEGG) pathway analysis was used. The top 30 KEGG pathways including “MicroRNAs in cancer,” “MAPK signaling pathway,” “Pathways in cancer,” “Signaling pathways regulating pluripotency of stem cells,” “Proteoglycans in cancer,” “Neurotrophin signaling pathway,” “Endocrine resistance,” “PI3K-Akt signaling pathway,” “Ras signaling pathway,” “FoxO signaling pathway,” “TNF signaling pathway,” “ErbB signaling pathway,” “HTLV-I infection,” “EGFR tyrosine kinase inhibitor resistance,” “Focal adhesion,” “Epstein-Barr virus infection,” “AGE-RAGE signaling pathway in diabetic complications,” “GnRH signaling pathway,” “Osteoclast differentiation,” “Hepatitis B,” “Prolactin signaling pathway,” “Estrogen signaling pathway,” “Protein processing in endoplasmic reticulum,” “Insulin resistance,” “mTOR signaling pathway,” “Herpes simplex infection,” “HIF-1 signaling pathway,” “Pancreatic cancer,” “Hepatitis C,” and “Dorso-ventral axis formation” (Fig. 4e). Among the top 30 KEGG pathways, “MAPK signaling pathway,” “PI3K-Akt signaling pathway,” “Ras signaling pathway,” and “HIF-1 signaling pathway” have been reported to be involved in angiogenesis. These results suggest that the presence of miRNA in hADSC-derived exosomes may explain their proangiogenic function to some extent.

### Identification of the key miRNAs in the proangiogenic activity of hADSC-derived exosomes

**Fig. 5:**
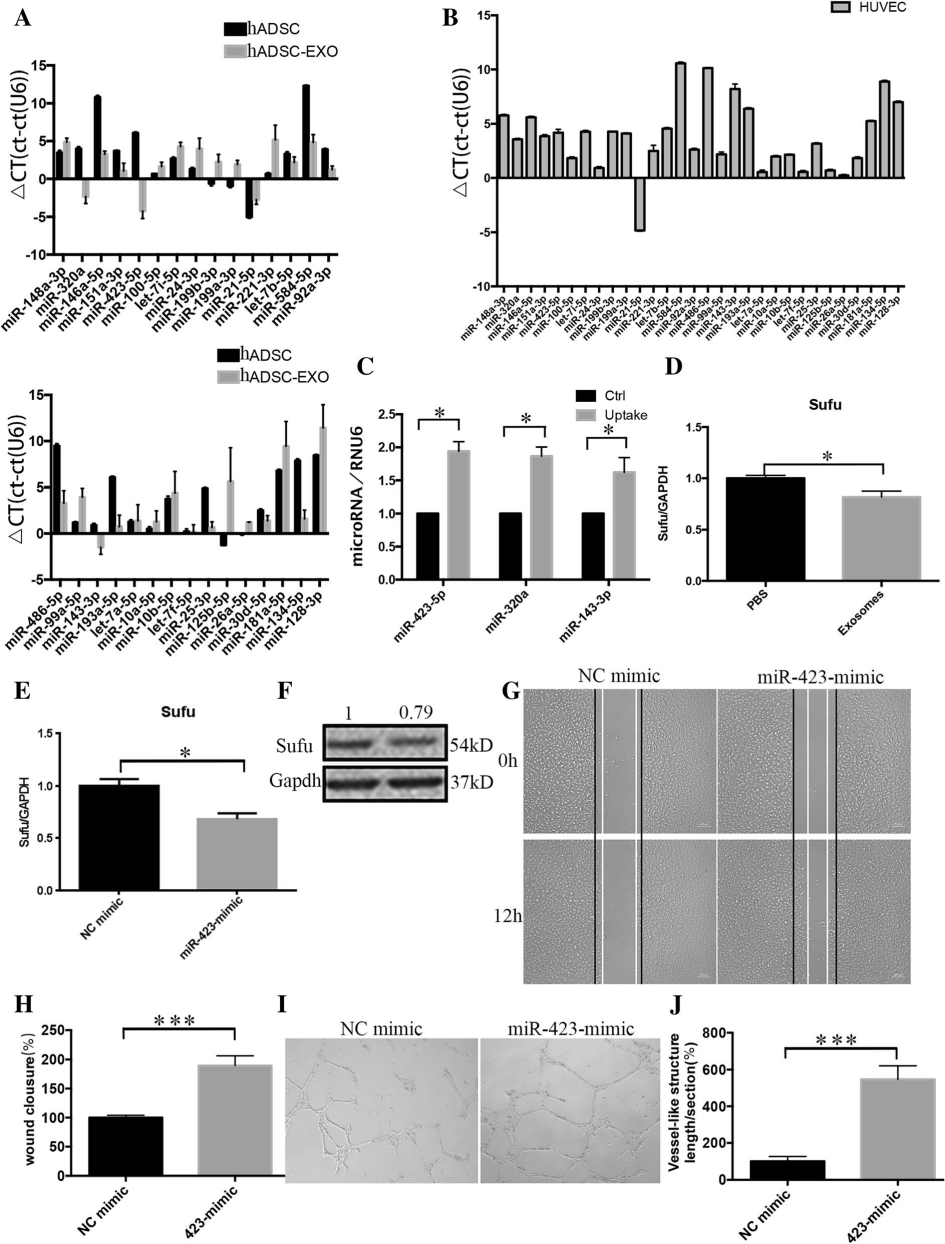
miR-423-5p may play a role in the proangiogenic activity of hADSC-derived exosomes. **a** RT-qPCR was used to determine the expression profile of the top 30 miRNAs in the exosomes and hADSCs. Delta CT was used to show the expression level of miRNAs relative to the internal reference RNU6: the larger the value, the lower the expression. **b** The basal expression level of the top30 miRNAs in HUVECs was determined using RT-qPCR. Delta CT was used to compare the expression level of miRNAs to the internal reference RNU6. (**c**) The changes of miR-423-5p, miR-320a and miR-143-3p after uptake of the exosomes by the HUVECs for 24 h was determined using RT-qPCR. Cells treated with PBS served as the control. **d** The changes in Sufu after HUVEC uptake of the exosomes for 48 h was determined using RT-qPCR. Cells treated with PBS served as the control. **e–f** Changes in the expression of Sufu in HUVECs 48 h after transfection with the miR-423-5p mimic were determined using RT-qPCR (**e**) and Western blotting (**f**). **g–h** Representative images of scratch assays recorded at 0 h and 12 h after transfection of the HUVECs with the miR-423-5p mimic. **i**–**j** Representative images of vessel-like formation assay recorded at 0 h and 12 h after plated the HUVECs transfected with the miR-423-5p mimic

**Fig. 6:**
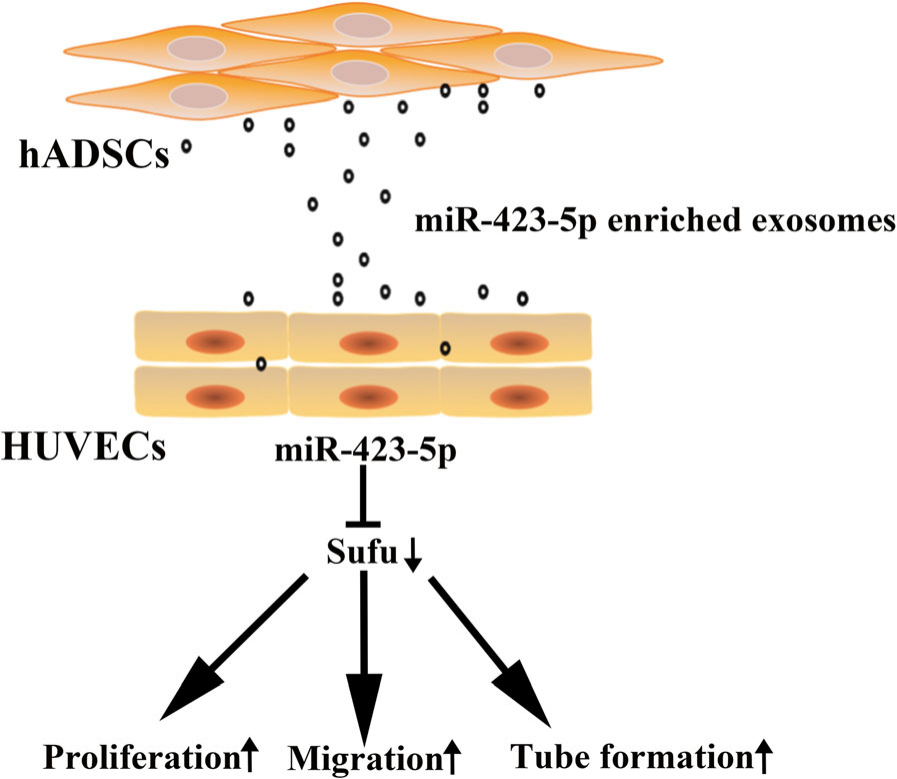
Schematic the proangiogenic activity of hADSC-derived exosomes

To confirm the miRNAs that were enriched in the exosomes, we use RT-qPCR to examine the expression profile of the top 30 most abundant miRNAs in exosomes and hADSCs in Table 1. As shown in Fig. 5a, the most enriched miRNAs were miR-423-5p, miR-320a, miR-143-3p, and miR-21-5p (Fig. 5a). In addition, we examined the baseline expression of these miRNAs in HUVECs. We found that miR-21-5p was highly expressed in endothelial cells HUVEC, which suggested that exogenous exosomal miR-21-5p was difficult to influence its endogenous expression level in endothelial cells (Fig. 5b). Therefore, we only tested the changes of miR-423-5p, miR-320a, and miR-143-3p after the uptake of the exosomes by HUVEC after 24 h. We found that the expression levels of miR-423-5p and miR-320a increased and there was a trend toward an increase in miR-143-3p in the HUVECs (Fig. 5c). In reviewing the literature, we found that miR-320a mainly plays a role in anti-angiogenesis [27], miR-143-3p showed proangiogenic and anti-angiogenic effects in different cases [28, 29], whereas miR-423-5p contributed to proangiogenesis [30]. In addition, Yang et al. found that exosomal miR-423-5p targets suppressor of fused (Sufu) to promote cell growth and metastasis [31]. Therefore, we speculated that miR-423-5p may play a key role in the proangiogenic effect of the hADSC-derived exosomes and may perform its function by downregulating Sufu expression. To verify our hypothesis, we determined the expression level of Sufu using RT-qPCR after HUVECs were cultured with exosome-conditional medium for 24 h. As shown in Fig. 5d, Sufu expression demonstrated a descending tendency compared to the PBS-treated HUVECs. To further verify whether the decrease of Sufu expression could be attributed to miR-423-5p, we transfected a miR-423-5p mimic into the HUVECs. The result showed that Sufu mRNA expression (Fig. 5e), as well as the protein (Fig. 5f) was inhibited. Subsequently, we used scratch and tube formation experiments to confirm the effect of miR-423-5p on the HUVECs. HUVECs that were transfected with the miR-423-5p mimic have an increased migration and tube formation capabilities than those transfected with an NC mimic (Fig. 5g–j). These results suggest that miR-423-5p is a key miRNA in the proangiogenic activity of hADSC-derived exosomes and plays a role by targeting Sufu (Fig. 6).

## Discussion

Recently, it has been discovered that cells release exosomes and use them as vehicles to share biologically active molecules with other cells in a paracrine and/or endocrine fashion [9]. Although any exosomes released by a cell are likely to carry cell type-specific membrane and cytosolic components, a number of common features are considered to be characteristic of exosomes. In particular, exosomes are between 50 and 150 nm in diameter and appear with a cup-shaped morphology [23] or rounded vesicles [32] under transmission electron microscopy. Apart from their morphology and size, their unique protein and lipid composition facilitate their identification. As a consequence of their endosomal origin, nearly all exosomes contain proteins that are involved in membrane transport and fusion (e.g., flotillin, Rab GTPases, Annexins), in multivesicular body (MVB) biogenesis (e.g., TSG101 and Alix), integrins and tetraspanins (e.g., CD9, CD63, CD81 and CD82), and so on [9] regardless of the cell type from which they originate. In this study, the exosomes were found to be rounded membrane vesicles with a diameter of 70–114 nm and expressed common exosomal proteins (CD9 and flotillin), which indicated that we successfully isolated high purity exosomes from the hADSC culture supernatant. Ultracentrifugation is a classic method for the isolation of exosomes. However, when the sample size is small, or when a large amount of exosomes is required for omics testing, the ultracentrifugation method is not suitable because of its low yield. Some commercial kits that may enhance the yields provide researchers with more choice. Royo et al. used miRNA profiling to compare the miRNAs expression of extracellular vesicles that were isolated using different methods and found that the methods of isolating exosomes have limited impact on the analysis of their miRNAs expression. Thus, to better satisfy the experimental requirements, we used two different methods (ultracentrifugation and commercial kit) to isolate exosomes from hADSCs. We used the commercial kit to obtain a sufficient amount of exosomes for the small RNA transcriptome sequencing.

MSCs show great promise in a wide array of therapeutic applications due mainly to their capacity to suppress immune and inflammatory reactions and initiate processes to repair damaged tissues. The secretion of exosomes that contain bioactive factors is thought to play an important role in the mechanisms of action for these clinically relevant functions. As one of the most promising MSCs, ADSCs have been reported to release exosomes that exhibit a proangiogenic potential. Eirin et al. demonstrated that porcine ADSC-derived exosomes are enriched with several types of miRNAs that target genes that modulate various cellular pathways including angiogenesis [33]. Furthermore, some proangiogenic miRNAs (e.g., miR-21) were observed to be enriched in the exosomes secreted by hADSCs. In this work, we demonstrated these miRNAs could be delivered into HUVECs via exosome internalization. Collectively, these observations suggest that hADSC-derived exosomes can promote angiogenesis as a paracrine signal. We further confirmed this speculation using some experiments in vitro. The results showed that hADSC-derived exosomes could effectively stimulate cell proliferation and migration after internalization by the HUVECs. More importantly, we promoted vessel-like formation by HUVECs in a dose-dependent manner, and the maximum activity (10 μg/mL) was comparable with that of 5% FBS. Thus, our research presents a new evidence in favor of the concept that hADSC-derived exosomes possess a potent proangiogenic activity.

To comprehensively explore the molecular mechanism by which hADSC-derived exosomes promote angiogenesis, we performed RNA-seq analysis to explore the miRNAs that were enriched in the exosomes. Overall, 845 miRNAs were detected in at least two of the samples, which accounted for 32.7% of the 2588 mature miRNAs in the miRbase (version21). Since the top 30 most abundant miRNAs accounted for an average of 84.6% of the total reads, we selected the 1119 gene targets that were predicted for these miRNAs and enrichmented these targets using GO and KEGG analysis. Among the KEGG pathways, “MAPK signaling pathway,” “PI3K-Akt signaling pathway,” “Ras signaling pathway,” and “HIF-1 signaling pathway” have been reported to be involved in angiogenesis. A previous study also discussed miRNA profiles from hADSCs cultured with FBS or platelet lysates [34]. Different from their work, we use GMP-level FBS substitute for cell culture, which is appropriate for clinical studies. As we all know, the culture conditions of stem cells have a great influence on their functions. This makes the essential difference exist between the two studies. And, this is the main reason for the significant differences in the data of small RNA sequencing between the two studies. Our study provides a rich resource for further Clinical Application Research of hADSC. However, the RNA-seq of exosomes also has certain limitations. When we verified the top 30 most abundant miRNAs using the gold standard RT-qPCR, we found that the Ct value for the RT-qPCR did not completely match the RPM for the RNA-seq. On the basis that the RNA-seq data was mainly used for prediction, we selected miRNAs that were highly expressed in both RT-qPCR and RNA-seq for further exploration. We found miR-423-5p was enriched in the hADSC-derived exosomes. Furthermore, uptake of the exosomes by the HUVECs clearly inhibited the expression of Sufu, which is a target of miR-423-5p that is predicted using miRDB and miRWalk and verified by Yang et al. [31]. Sufu is a negative regulator of cells’ biological function, and it has been well documented in various cells that downregulation of Sufu can increase cell proliferation and migration ability [35]. Moreover, exosomal miR-423-5p has been reported to promote tumor growth and metastasis by targeting Sufu [31]. In our study, the miR-423-5p mimic could enhance the migration and tube formation ability of HUVECs by downregulating the expression of Sufu. This indicated that miR-423-5p may be the key miRNAs in the hADSC-derived exosomes and may mediate its proangiogenic activity by targeting Sufu. To our knowledge, it is the first time to demonstrate that miR-423-5p has been demonstrated to promote angiogenesis through the Sufu signaling pathway under normal conditions.

Exosomes are heterogeneous in nature and in content, which enables them to deliver multiple molecules that have a synergistic effect on angiogenesis. However, this heterogeneity also creates some problems regarding the standardization of the exosome preparation process for eventual therapeutic application. Thus, it is important to identify and categorize the active molecules carried by these exosomes, because this may not only contribute to explaining the mechanism of action of the native exosomes but also allow the generation of engineered exosomes that are enriched with potential angiogenic molecules that would facilitate the standardization of exosomes. In our study, we performed RNA-seq using exosomes isolated from media in which FBS was replaced with UltraGRO-Advanced (GMP Grade). This allowed our research to more realistically reflect the active substances that are encapsulated in exosomes during clinical application.

## Conclusion

In this study, we purified exosomes from hADSCs and described their proangiogenic activity in vitro. We further examined the miRNAs expression in the exosomes using RNA sequencing. More importantly, we demonstrated for the first time that miR-423-5p was a key active molecule in the proangiogenic activity of exosomes. This provides strong support for the concept that hADSC-derived exosomes or similar engineered exosomes could potentially be exploited as a therapeutic tool for regenerative medicine.

## Additional file

Additional file 1: **Figure S1.** Particle size/relative intensity 3D plot of exosomes by using nanosight technology, relative to Fig. 2C. **Figure S2.** Image of primary HUVEC, relative to Fig. 2D. **Figure S3.** Representative photos of the HUVEC uptake of PKH26-labeled exosomes (10 μg/ml, 5 μg/ml, 2.5 μg/ml) at 3 h, 6 h, 12 h, and 21 h. Bar = 10 μm, relative to Fig. 2E. (ZIP 2100 KB)

## Abbreviations

BCA: Bicinchoninic acid
BP: Biological processes
Ct: Threshold cycle
EC: Endothelial cell
EdU: 5-Ethynyl-2′-deoxyuridine
FBS: Fetal bovine serum
GO: Gene Ontology
hADSCs: Human adipose-derived stem cells
HBSS: Hank’s balanced salt solution
HUVECs: Human umbilical vein endothelial cells
KEGG: Kyoto encyclopedia of genes and genomes
MSCBM: Mesenchymal Stem Cell Basal Medium
MSCs: Multipotent stromal cells
MVB: Multivesicular body
NTA: NanoSight analysis
PBS: Phosphate-buffered saline
RPM: Reads per million
RT: Room temperature
Sufu: Suppressor of fused

## References

[1] da Silva Meirelles L, Chagastelles PC, Nardi NB (2006). Mesenchymal stem cells reside in virtually all post-natal organs and tissues. J Cell Sci.

[2] Keating A (2012). Mesenchymal stromal cells: new directions. Cell Stem Cell.

[3] Mendicino M, Bailey AM, Wonnacott K, Puri RK, Bauer SR (2014). MSC-based product characterization for clinical trials: an FDA perspective. Cell Stem Cell.

[4] Xu F, Liu J, Deng J, Chen X, Wang Y, Xu P (2015). Rapid and high-efficiency generation of mature functional hepatocyte-like cells from adipose-derived stem cells by a three-step protocol. Stem Cell Res Ther.

[5] Jiang M, Zheng C, Shou P, Li N, Cao G, Chen Q (2016). SHP1 regulates bone mass by directing mesenchymal stem cell differentiation. Cell Rep.

[6] Zhou J, Zhao L (2016). Multifunction Sr, Co and F co-doped microporous coating on titanium of antibacterial, angiogenic and osteogenic activities. Sci Rep.

[7] Phinney DG, Prockop DJ (2007). Concise review: mesenchymal stem/multipotent stromal cells: the state of transdifferentiation and modes of tissue repair--current views. Stem Cells.

[8] Lavoie JR, Rosu-Myles M (2013). Uncovering the secretes of mesenchymal stem cells. Biochimie..

[9] Raposo G, Stoorvogel W (2013). Extracellular vesicles: exosomes, microvesicles, and friends. J Cell Biol.

[10] van der Pol E, Boing AN, Harrison P, Sturk A, Nieuwland R (2012). Classification, functions, and clinical relevance of extracellular vesicles. Pharmacol Rev.

[11] Valencia K, Lecanda F (2016). Microvesicles: isolation, characterization for in vitro and in vivo procedures. Methods Mol Biol.

[12] Kishore R, Khan M (2016). More than tiny sacks: stem cell exosomes as cell-free modality for cardiac repair. Circ Res.

[13] Nakamura Y, Miyaki S, Ishitobi H, Matsuyama S, Nakasa T, Kamei N (2015). Mesenchymal-stem-cell-derived exosomes accelerate skeletal muscle regeneration. FEBS Lett.

[14] Baglio SR, Pegtel DM, Baldini N (2012). Mesenchymal stem cell secreted vesicles provide novel opportunities in (stem) cell-free therapy. Front Physiol.

[15] Bruno S, Collino F, Deregibus MC, Grange C, Tetta C, Camussi G (2013). Microvesicles derived from human bone marrow mesenchymal stem cells inhibit tumor growth. Stem Cells Dev.

[16] Sahoo S, Klychko E, Thorne T, Misener S, Schultz KM, Millay M (2011). Exosomes from human CD34(+) stem cells mediate their proangiogenic paracrine activity. Circ Res.

[17] Harp D, Driss A, Mehrabi S, Chowdhury I, Xu W, Liu D (2016). Exosomes derived from endometriotic stromal cells have enhanced angiogenic effects in vitro. Cell Tissue Res.

[18] Kholia S, Ranghino A, Garnieri P, Lopatina T, Deregibus MC, Rispoli P (2016). Extracellular vesicles as new players in angiogenesis. Vasc Pharmacol.

[19] Kane NM, Thrasher AJ, Angelini GD, Emanueli C (2014). Concise review: MicroRNAs as modulators of stem cells and angiogenesis. Stem Cells.

[20] Landskroner-Eiger S, Moneke I, Sessa WC (2013). miRNAs as modulators of angiogenesis. Cold Spring Harbor Perspect Med.

[21] Poliseno L, Tuccoli A, Mariani L, Evangelista M, Citti L, Woods K (2006). MicroRNAs modulate the angiogenic properties of HUVECs. Blood..

[22] Phinney DG, Di Giuseppe M, Njah J, Sala E, Shiva S, St Croix CM (2015). Mesenchymal stem cells use extracellular vesicles to outsource mitophagy and shuttle microRNAs. Nat Commun.

[23] Thery C, Amigorena S, Raposo G, Clayton A. Isolation and characterization of exosomes from cell culture supernatants and biological fluids. Curr Protoc Cell Biol. 2006;30(1):3.22.1–3.22.29. 10.1002/0471143030.cb0322s30 18228490

[24] Mulcahy LA, Pink RC, Carter DR. Routes and mechanisms of extracellular vesicle uptake. J Extracellular Vesicles. 2014;3(1):24641. 10.3402/jev.v3.24641 PMC4122821 25143819

[25] Svensson KJ, Christianson HC, Wittrup A, Bourseau-Guilmain E, Lindqvist E, Svensson LM (2013). Exosome uptake depends on ERK1/2-heat shock protein 27 signaling and lipid raft-mediated endocytosis negatively regulated by caveolin-1. J Biol Chem.

[26] Carmeliet P, Jain RK (2011). Molecular mechanisms and clinical applications of angiogenesis. Nature..

[27] Sun JY, Zhao ZW, Li WM, Yang G, Jing PY, Li P (2017). Knockdown of MALAT1 expression inhibits HUVEC proliferation by upregulation of miR-320a and downregulation of FOXM1 expression. Oncotarget..

[28] Jin YP, Hu YP, Wu XS, Wu YS, Ye YY, Li HF (2018). miR-143-3p targeting of ITGA6 suppresses tumour growth and angiogenesis by downregulating PLGF expression via the PI3K/AKT pathway in gallbladder carcinoma. Cell Death Dis.

[29] Lawson J, Dickman C, MacLellan S, Towle R, Jabalee J, Lam S (2017). Selective secretion of microRNAs from lung cancer cells via extracellular vesicles promotes CAMK1D-mediated tube formation in endothelial cells. Oncotarget..

[30] Li S, Zeng A, Hu Q, Yan W, Liu Y, You Y (2017). miR-423-5p contributes to a malignant phenotype and temozolomide chemoresistance in glioblastomas. Neuro-Oncol.

[31] Yang H, Fu H, Wang B, Zhang X, Mao J, Li X (2018). Exosomal miR-423-5p targets SUFU to promote cancer growth and metastasis and serves as a novel marker for gastric cancer. Mol Carcinog.

[32] Lasser C, Eldh M, Lotvall J. Isolation and characterization of RNA-containing exosomes. J Visualized Exp. 2012;59:e3037. 10.3791/3037 PMC3369768 22257828

[33] Eirin A, Riester SM, Zhu XY, Tang H, Evans JM, O'Brien D (2014). MicroRNA and mRNA cargo of extracellular vesicles from porcine adipose tissue-derived mesenchymal stem cells. Gene..

[34] Baglio SR, Rooijers K, Koppers-Lalic D, Verweij FJ, Perez Lanzon M, Zini N (2015). Human bone marrow- and adipose-mesenchymal stem cells secrete exosomes enriched in distinctive miRNA and tRNA species. Stem Cell Res Ther.

[35] Lee DY, Deng Z, Wang CH, Yang BB (2007). MicroRNA-378 promotes cell survival, tumor growth, and angiogenesis by targeting SuFu and Fus-1 expression. Proc Natl Acad Sci U S A.

