# Supplementary figures and images for: Exosomal miR-423-5p mediates the proangiogenic activity of human adipose-derived stem cells by targeting Sufu
Source: Stem Cell Res Ther. 2019 Mar 21;10:106. doi: 10.1186/s13287-019-1196-y (PMC6429803; doi:10.1186/s13287-019-1196-y)

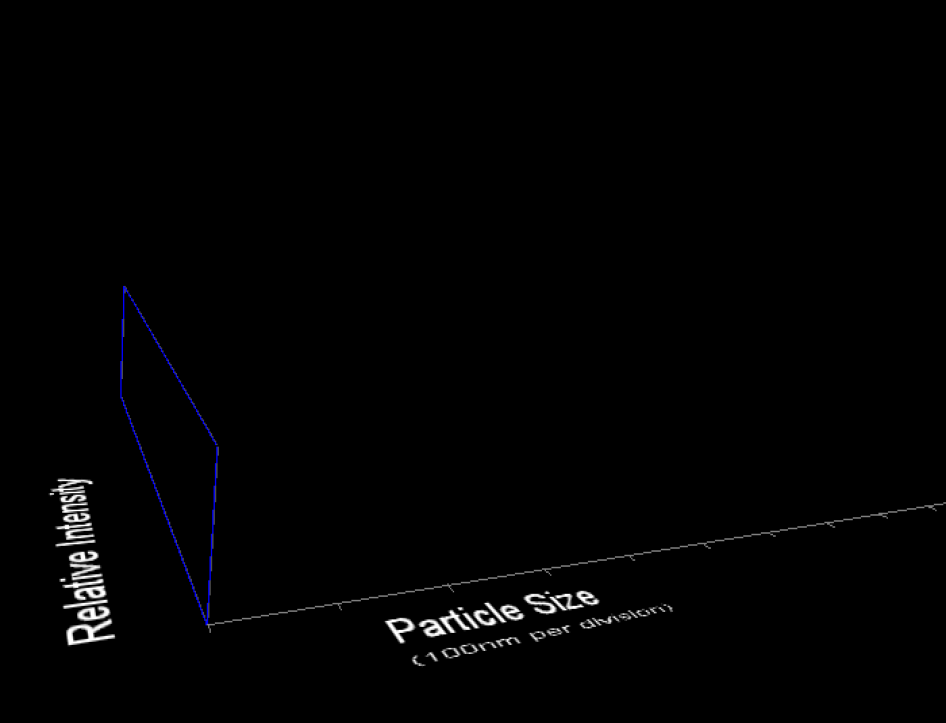

Supplement: Supplementary file 1 — Figure S1. Particle size/relative intensity 3D plot of exosomes by using nanosight technology, relative to Fig. 2C. Figure S2. Image of primary HUVEC, relative to Fig. 2D. Figure S3. Representative photos of the HUVEC uptake of PKH26-labeled exosomes (10 μg/ml, 5 μg/ml, 2.5 μg/ml) at 3 h, 6 h, 12 h, and 21 h. Bar = 10 μm, relative to Fig. 2E. (ZIP 2100 KB) [file 13287_2019_1196_MOESM1_ESM.zip › 13287_2019_1196_MOESM1_ESM/Figure S1.tif]

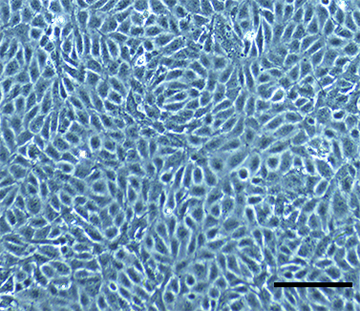

Supplement: Supplementary file 1 — Figure S1. Particle size/relative intensity 3D plot of exosomes by using nanosight technology, relative to Fig. 2C. Figure S2. Image of primary HUVEC, relative to Fig. 2D. Figure S3. Representative photos of the HUVEC uptake of PKH26-labeled exosomes (10 μg/ml, 5 μg/ml, 2.5 μg/ml) at 3 h, 6 h, 12 h, and 21 h. Bar = 10 μm, relative to Fig. 2E. (ZIP 2100 KB) [file 13287_2019_1196_MOESM1_ESM.zip › 13287_2019_1196_MOESM1_ESM/Figure S2.tif]

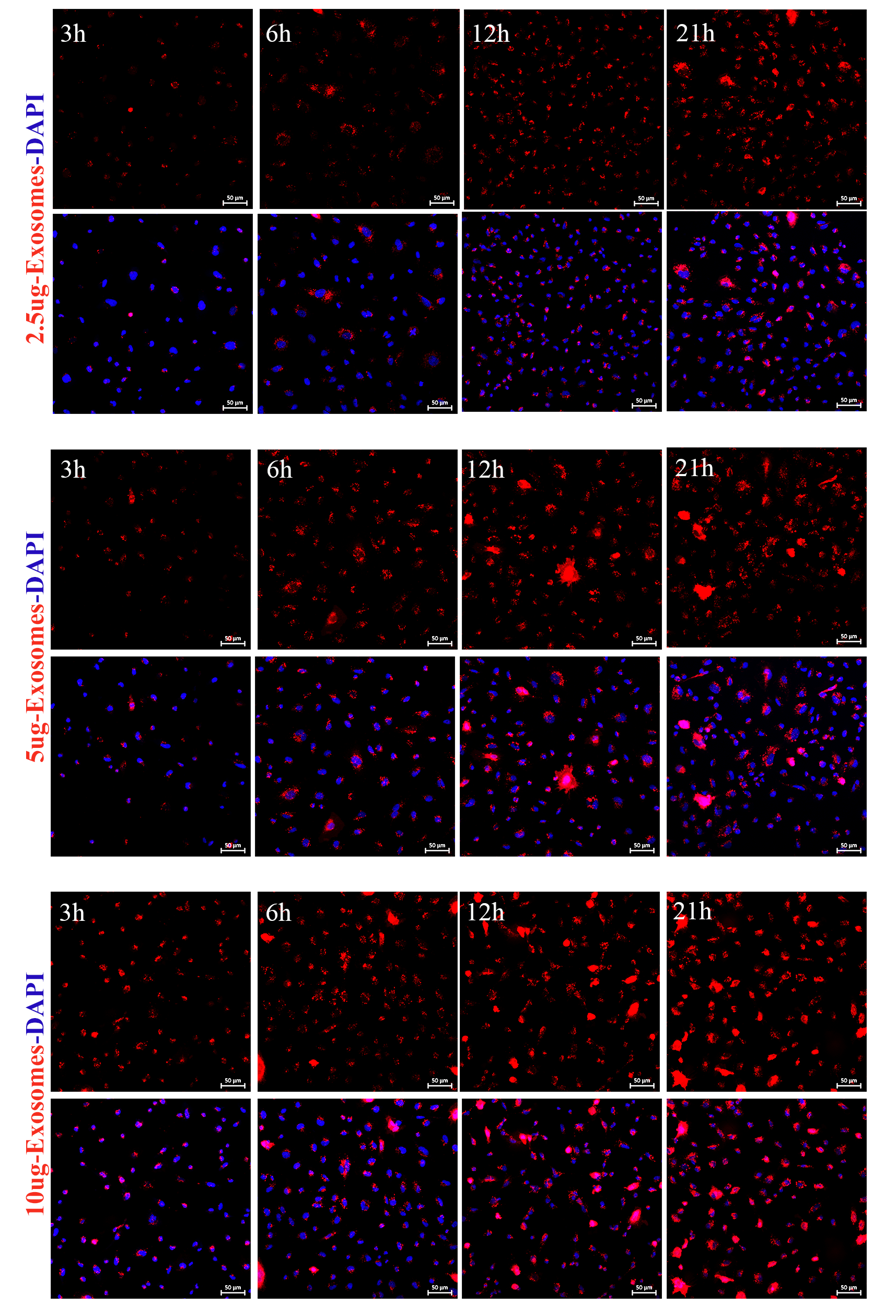

Supplement: Supplementary file 1 — Figure S1. Particle size/relative intensity 3D plot of exosomes by using nanosight technology, relative to Fig. 2C. Figure S2. Image of primary HUVEC, relative to Fig. 2D. Figure S3. Representative photos of the HUVEC uptake of PKH26-labeled exosomes (10 μg/ml, 5 μg/ml, 2.5 μg/ml) at 3 h, 6 h, 12 h, and 21 h. Bar = 10 μm, relative to Fig. 2E. (ZIP 2100 KB) [file 13287_2019_1196_MOESM1_ESM.zip › 13287_2019_1196_MOESM1_ESM/Figure S3.tif]
